# P28 Evaluation of one in-house and three commercial phenotypic tests for detection of KPC, NDM and OXA-48-like carbapenemase in *Klebsiella pneumoniae*: is routine detection possible?

**DOI:** 10.1093/jacamr/dlad143.032

**Published:** 2024-01-03

**Authors:** Arwa AlRujaibi, Meher Rizvi, Zaaima Al Jabri

**Affiliations:** Department of Microbiology and Immunology, College of Medicine and Health Sciences, Sultan Qaboos University, Muscat, Oman; Department of Microbiology and Immunology, College of Medicine and Health Sciences, Sultan Qaboos University, Muscat, Oman; Department of Microbiology and Immunology, College of Medicine and Health Sciences, Sultan Qaboos University, Muscat, Oman

## Abstract

**Background:**

Phenotypic characterization of the OXA-48-like carbapenemase XDR and pandrug-resistant (PDR) *Klebsiella pneumoniae* in clinical isolates is essential for making informed empirical decisions and critical for strengthening antimicrobial stewardship programmes. This study focused on assessing one in-house and three commercial phenotypic tests for detection of KPC, NDM and OXA-48-like carbapenemases in an attempt to study the feasibility of making detection of OXA-48-like carbapenemases a routine activity of a diagnostic microbiology laboratory.

**Methods:**

A total of 40 representative non-duplicate XDR and PDR clinical *Klebsiella pneumoniae* isolates were screened on the basis of susceptibility profile, resistance to piperacillin/tazobactam; susceptible, intermediate, or resistant to third generation cephalosporins; elevated MIC for meropenem. These isolates were then subjected to one in-house test (OXA-48 disc test) and three commercial tests: Xpert® Carba-R (Cepheid), MASTDISCS® Combi Carba Plus (D73C), Immunochromatographic assay (ICT); KPC/IMP/NDM/OXA-48 Combo test (LFIA), Medomics.

**Results:**

Xpert ®Carba-R identified OXA-48 in 62.5% (*n*=25) isolates, NDM in 17.5% (*n*=7), KPC+NDM co-carriage in 15% (*n*=6) and KPC in one isolate. Both Xpert® Carba-R and ICT were considered as possible gold standards and the outcomes of the other tests were compared with these two. With Xpert® Carba-R as the gold standard, high sensitivity of 96.15% was detected in OXA-48-like isolates, followed by NDM (92.85%) and NDM+KPC (85.7%). The lowest sensitivity was seen with the OXA-48 disc test (48% in OXA-48, 53.8% in NDM) followed by D73C test (64% in OXA-48, 38.46% in NDM). These two tests could not detect co- carriage of NDM and KPC in six isolates, although in two cases one of the two was detected. Specificity was high among all the tests (96.29%–93.33%). With ICT as the gold standard, 100% sensitivity was reported for NDM and KPC isolates, followed by OXA-48 and KPC+NDM (96% and 83.33%, respectively). The specificity was higher with ICT (99%) than Xpert® Carba-R (96.29%) as in detection of co- carriage of NDM and KPC. In terms of cost ICT ($16 per test) was much cheaper than Xpert® Carba-R($52 per test).

**Conclusions:**

We recommend immunochromatographic assay (ICT) as an excellent tool for detection of carbapenemases (KPC/IMP/NDM/OXA-48). OXA-48 disc test and MASTDISCS® Combi Carba plus (D73C) cannot be considered as good standard since they could not detect co-carriage of NDM and KPC and had lower sensitivity.

